# The SIMAC study: A randomized controlled trial to compare the effects of resistance training and aerobic training on the fitness and body composition of Colombian adolescents

**DOI:** 10.1371/journal.pone.0248110

**Published:** 2021-04-20

**Authors:** Daniel Dylan Cohen, Gavin R. Sandercock, Paul Anthony Camacho, Johanna Otero-Wandurraga, Sandra Milena Pinzon Romero, Rocío del Pilar Martínez Marín, Camilo Andrés Villamizar Sierra, Javier Carreño, Jason Moran, Patricio Lopez-Jaramillo

**Affiliations:** 1 Masira Research Institute, Faculty of Health Sciences, Universidad de Santander (UDES), Bucaramanga, Colombia; 2 Sports Science Center (CCD), Colombian Ministry of Sport (Mindeporte), Colombia; 3 Essex University, Colchester, United Kingdom; 4 Fundación Oftalmologica de Santander (FOSCAL), Bucaramanga, Colombia; 5 Universidad Autónoma de Bucaramanga (UNAB), Bucaramanga, Colombia; 6 Department of Human Movement, Universidad Autonoma de Manizales (UAM), Manizales, Colombia; Edinburgh Napier University, UNITED KINGDOM

## Abstract

The aim of this study was to evaluate the impact on muscle strength, aerobic fitness and body composition, of replacing the physical education (PE) class of Colombian adolescents with resistance or aerobic training. 120 tanner stage 3 adolescents attending a state school were randomized to resistance training, aerobic training, or a control group who continued to attend a weekly 2- hour PE class for 16 weeks. The resistance training and aerobic training groups participated in twice weekly supervised after-school exercise sessions of < 1 hour instead of their PE class. Sum of skinfolds, lean body mass (bioelectrical impedance analysis), muscular strength (6 repetition maximum (RM)) bench press, lateral pulldown and leg press) and estimated cardiorespiratory fitness (multistage 20 meter shuttle run) were assessed at pre and post intervention. Complete data were available for n = 40 of the resistance training group, n = 40 of the aerobic training group and n = 30 PE (controls). Resistance training attenuated increases in sum of skinfolds compared with controls (d = 0.27, [0.09–0.36]). We found no significant effect on lean body mass. Resistance training produced a positive effect on muscle strength compared with both controls (d = 0.66 [.49-.86]) and aerobic training (d = 0.55[0.28–0.67]). There was a positive effect of resistance training on cardiorespiratory fitness compared with controls (d = 0.04 [-0.10–0.12]) but not compared with aerobic training (d = 0.24 [0.10–0.36]). Replacing a 2-hour PE class with two 1 hour resistance training sessions attenuated gains in subcutaneous adiposity, and enhanced muscle strength and aerobic fitness development in Colombian youth, based on a median attendance of approximately 1 session a week. Further research to assess whether adequate stimuli for the development of muscular fitness exists within current physical education provision is warranted.

## Introduction

International guidelines recommend that youth should partake in at least 60 min of moderate-to-vigorous physical activity each day incorporating three bouts of muscle strengthening activities [1]. Strength activities are an intrinsic element of current physical activity guidelines for adults older adults and young people [1], based on growing evidence that muscular strength is associated with better cardiometabolic health throughout the lifecycle [2–5]. The proportion of young people who meet current guidelines for strength activity is much lower however, than for aerobic exercise [6].

The PURE study of >135,000 adults from 21 countries found that strength decreased cardiovascular risk and all-cause mortality [5]. The associated burden of disease related to low muscle strength has been estimated at $18.5 Billion in the US [7]. Importantly, from a public health perspective, muscle strength is lower in adults living in low-middle income countries compared to high-income country populations [8]. This low-middle income country “strength deficit” appears to have its origins in earlier life as strength tracks from childhood [9] and lower birthweight, an indicator of poorer in uterine nutrition and more common in low-middle income countries, are associated with lower muscle strength throughout life [10]. Indeed, we have reported that children in Colombia (a middle-income country), particularly those from low-to-middle socio-economic households [11, 12], have lower muscle strength compared with that reported in high-income countries [13].

Activities to increase muscle strength, such as resistance training, provide a powerful stimulus for strength development [14], and may therefore be of particular importance in young people in low-middle income country populations. Little is known however regarding the implementation and outcomes of resistance training interventions in low-middle income countries. The majority of studies have been conducted in high-income countries [15, 16], and a recent systematic review of resistance training programs in schools showed that only 1 of 11 studies was implemented in an low-middle income country [15]. School represents the ideal setting for the delivery of resistance training as it provides opportunities to introduce students to novel forms of activity they might not be exposed to outside of school [17]. Unfortunately, resistance training is an underutilized tool in the school setting, and the apparently inadequate stimulus school aged children typically receive for muscular fitness is evident from their declining strength levels [18, 19]. Exposing school age children to strengthening exercise could be an ideal opportunity to enhance current and future health [6].

While the objective of exercise interventions in youth is often to combat overweight and obesity- as approximately 18% of Colombian 10–17 year old are overweight or obese [20]- a much lower prevalence than in many high income countries [21], our interest in resistance training is instead to assess its potential to improve muscular fitness, cardiorespiratory fitness and body composition, as well as physical literacy.

Therefore, the principal aim of the present study was to examine the effects of a 16 week supervised resistance training intervention on health related physical fitness in Colombian school children when compared to both physical education alone and aerobic training control conditions. In this way, we sought to isolate the impact of resistance training on measures of physical fitness and body composition and assess the practical viability of implementing such an exercise program in place of PE classes in a resource constrained low-middle income country.

## Materials and methods

### Study population and design

The study was approved by the University of Santander Ethics Committee and the principal of CEDECO, a mixed sex state primary and secondary school in Piedecuesta, Santander, Colombia, and was conducted in accordance with the principles of the Helsinki declaration. The study was part one of a clinical trial (NCT03779737) SIMAC: Fuerza muscular y capacidad aeróbica relación SImbiótica en escolares con bajo peso al nacer y riesgo MetAbóliCo (Symbiotic relationship between muscular strength and aerobic capacity, metabolic risk and low birthweight in schoolchildren). We recruited by inviting all students aged between 13–17 and their parents to presentations given by the investigators at the school to outline the study. For those students who were interested in participating and their parent or guardian gave their assent, we obtained written informed consent from the parent/guardian. These students completed a questionnaire to determine if they had a physical limitation or medical condition which would prevent them from, or put them at risk from, performing the evaluations or the training program. Individuals who were currently involved in a structured exercise or sports program were also excluded. Those who were eligible based on these criteria were then invited to attend a medical examination. To confirm volunteers were above Tanner stage 3, they first underwent maturational assessment with a trained nurse. We excluded those below tanner stage 3 in order to ensure a maturationally less heterogenous group of participants. Eligible volunteers were then invited to participate and were randomly allocated to progressive resistance training (“*resistance*”) or aerobic training (“*aerobic*”, or normal PE class (“*control*”) groups. Children and parents were informed that they were free to withdraw from the study at any time with no penalty.

### Measures

After the medical evaluation, but prior to randomization, participants eligible for the study underwent anthropometric and physical fitness evaluations at the school. These evaluations were repeated at the end of the program and were carried out by qualified physiotherapists, doctors and trained research assistants (physiotherapy students).

Height and weight were measured in light clothing without shoes using a portable digital scale (Tanita, Arlington Heights, IL, USA) to the nearest 0.1 kg, and height was measured to the nearest 0.1 cm using a portable stadiometer (SECA wall-mounted stadiometer 206, Germany). Lean body mass was estimated from bioelectrical-impedance (Ironman BC– 554, Tanita, Arlington Heights, IL, USA) with each child measured either in a morning or afternoon session with their post test repeated during the same part of the day to reduce potential variability in impedance related to differences in food intake, activity or hydration. Skinfold thickness (Harpenden, Baty International, Burgess Hill, UK) was measured at seven sites (triceps, biceps, subscapular, suprailiac, abdominal, thigh and calf) [22] by a physiotherapist with several years of experience in this assessment. The total skinfold thickness (in mm) was summed as an index of subcutaneous adiposity. We quantified changes in adiposity using sum of skinfolds instead of providing an estimate of fat mass or % fat using this data or that from bioelectrical impedance. We elected to do so to evaluate relative change in a direct measure of subcutaneous fat without depending on the appropriateness for the present population of the assumptions inherent in formulas for conversion of this value to total % fat or fat mass [23].

We measured six repetition maximum (6 RM) of chest- press, lateral-pulldown and leg-press and the sum in kg was used as a composite measure of strength. We assessed 6 RM, by first demonstrating the exercise to a small group of participants and following a warm-up including mobility exercises and dynamic stretching. Each participant then performed two sets that were deliberately submaximal in load and repetitions to familiarize with the appropriate technique and range of motion required. We then estimated a load which, based on their feedback on the level of difficulty in performing the practice sets, we estimated would challenge them. Typically, the participant could perform 6 repetitions with the full range of motion in this third set, and further load was added in a subsequent set, but if they could not achieve the full range of motion we reduced the weight for them in a subsequent set. Three minutes rest was given between each subsequent set until we identified the weight at which they could complete no more than 6 repetitions with proper form through the full range of motion [24]. We estimated cardiorespiratory fitness by assessing number using the number of levels completed in the 20 meter shuttle-run test [25].

### Treatment conditions

Our primary interest was in the effects of resistance compared to curriculum PE class. However, to ensure that the potential adaptations observed could be ascribed specifically to stimulus of the resistance program and not confounded by the influence of participating in a novel and more closely supervised program than PE represents (i.e. 1 teacher to 30–40 students), a second intervention group who participated in an aerobic program at the same site, within the same hours provided a “second control group”. Intervention groups were prescribed 16 weeks of twice-weekly supervised aerobic or resistance activity performed on non-consecutive days. The control group continued to participate in weekly 2 hour PE class of 120 min, and were also asked to not begin a new structured exercise program for the period of the study.

The structure of the schoolday within Colombia’s state education system dictates that students either attend all classes in either a single morning session (6.15 AM to 12.15 PM) or a single afternoon session (12.30 to 6.30 PM). Typically, students swap between mornings and afternoons on an annual basis. This schedule and a lack of space at the school meant that the program had to be offered offsite either before or after school dependent on whether they attended morning or afternoon classes.

Training sessions were conducted in groups of no more than 10 participants and were led by qualified physiotherapists, with the additional support of physiotherapy students who were trained prior to the study. In Colombia, physiotherapy degrees have a substantial practical exercise prescription component for programming in healthy individuals, including in youth. These degrees contain much of the content of a sports science program and as such, the program was appropriately supervised by such graduates. The supervision involved setting the prescribed load, monitoring, and where appropriate, correcting exercise technique, encouraging effort and monitoring and recording number of sets, repetitions and rest period between exercises.

Resistance and aerobic participants were collected from school, transported to the site in a chartered bus service (10–15 minutes) and returned after each scheduled session. All exercise sessions commenced with a warm-up. The resistance training warm up began with 1 set of 15 reps of mobility exercises for the shoulder, elbow, wrist, trunk, hops, knee and ankle joints. Warm-up continued with static stretches for the major muscle groups of the upper and lower body each, held for 30s and a 10 minute run on flat terrain including dynamic stretches. Cooldown after both resistance and aerobic sessions involved a 10-minute light jog during which joint mobility, and dynamic and static stretches were performed. The aerobic warm-up and cooldowns had the same content but used a 5 instead of a 10-minute jog.

### Resistance training

Each participant’s 6 repetition maximum (6 RM) was used to estimate their 1 RM using the Brzycki formula (%1RM = 102.78–2.78 * repetitions) [26]. This was used to determine the load corresponding to the prescribed intensity (% of 1RM) for the first week of training. The resistance training program was informed by accepted guidelines for resistance training in youth [14, 27] and involved a twice a week progressive periodized program including a variety of exercises for all major muscle groups using resistance machine exercises (Sport fitness, Medellín, Colombia) and free weights (bars and dumbbells), medicine balls, Bosu and Swiss balls. Participants performed between 2 and 4 sets of an exercise with between 30–120 seconds rest between sets. An overview of the exercises integrated into the resistance training program are shown in Table 1, with specific exercise selection based on guidelines [14, 27] and practical considerations such as what equipment was available, efficient use of time with that equipment, and variety to reduce monotony of the program.

**Table 1:** Overview of exercises used in resistance training program.

Multigym (machine) exercises	Free-weights	Bodyweight exercises	Functional/Proprioception
Pec fly Triceps push down (elbow extension) Pulldown-pronated grip Pulldown-supine grip Seated row Chest press Shoulder press Leg press Calf raise (plantar flexion) Knee extension Leg curl (knee flexion) Abdominal crunch (trunk flexion)	DB pullover BB biceps curl DB front rasie (shoulder flexion) DB lateral raise (shoulder flexion) DB/BB bench press Military press BB/DB Row BB Squat DB walking lunge DB dynamic lunge BB lunge Step ups with BB on shoulder Sumo squat BB Triceps extension	Press ups/half press- ups Lumbar extension Abdominal rotation Abdominal crunch	Bilateral squat on Bosu Unilateral squat on Bosu Abdominal crunch (trunk flexion) on stability ball Medicine ball throw

DB = Dumbell, BB = Barbell.

Overall, intensity was increased across the 16 weeks such that during weeks 1–5; 16–20 RM with 30 seconds rest between sets, in weeks 6–10; between 8–14 RM, with between 30–120 seconds inter-set rest, and in weeks 11–16; between 6 and 12 RM with 30–120 seconds inter-set rest. For the multigym exercises (Table 1) load was adjusted by re-estimated participant’s 1 RM according to the load and repetitions performed during the last session of the training block, whereas exercises in the free-weight and functional/proprioceptive categories were progressed in a more informal way by estimating adding load to try and maintain participants within the target repetition range.

### Aerobic training

During the aerobic sessions heart rate (HR) of each participant was monitored continuously (MyZone MZ-3 HR monitors, Nottingham, UK) with data live streamed onto a TV screen. The instructor could observe the HR’s of the whole group and determine if a participant was in the correct intensity zone, encouraging greater effort in those below their prescribed target zone and vice versa, as per target zone appropriate to the phase of the program.

The intensity of the sessions was progressively increased from moderate to vigorous across the course of the intervention as shown in Table 3. In order to determine the HR range which corresponded to the prescribed intensity, we estimated maximum heart rate according to the Astrand’s formula (HR max = 216.6 –(0.84 x age)) [26]. Intensity and duration were progressed across the 16-week program as follows: weeks 1–3; 20 minutes at 65–75% of estimated max HR; weeks 4–6; 30 mins at 65–75%, weeks 7–12; 30 minutes at 70–80%, weeks 13–16; 35 minutes at 70–80%. The principal activities used in aerobic were variations in straight line and multi-directional jogging/running, with obstacles such as cones, hoops and speed ladders. Sessions also included step aerobics and sports orientated sessions incorporating balls.

### Statistical analyses and data treatment

Data analysis was performed with Statistical Package for the Social Sciences (SPSS, version 25, SPSS Inc., Chicago, Ill, USA). The analysis included participants who attended both assessments with complete data for each outcome measure. We calculated means and standard deviations (SD) for all continuous measures (Table 1).

To evaluate the efficacy of the resistance program we compared post-test means of all outcome measures. We used Analysis of Covariance (ANCOVA) group with condition as the fixed factor including pre-intervention test values as a covariate to adjust for between-group differences at baseline and potential regression to the mean. The magnitude of differences between the estimated marginal means was expressed as Cohen’s *d* with CI (95% confidence intervals). We interpreted effect sizes as small (*d* = 0.2–0.5), moderate (*d* = 0.5–0.8) and large (*d* = > 0.8).

**Fig 1:**
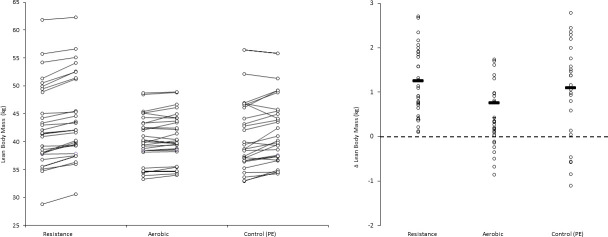
**Left panel: Lean body mass values (kg) pre- and post- intervention for each participant, and group means.** Right panel: Individual participant and mean within-group post-intervention lean body mass difference (kg). Pairwise effect sizes for within-group difference in lean body mass: Resistance; *d =* 0.18, aerobic; *d =* 0.15, PE (control); *d* = 0.17.

**Fig 2:**
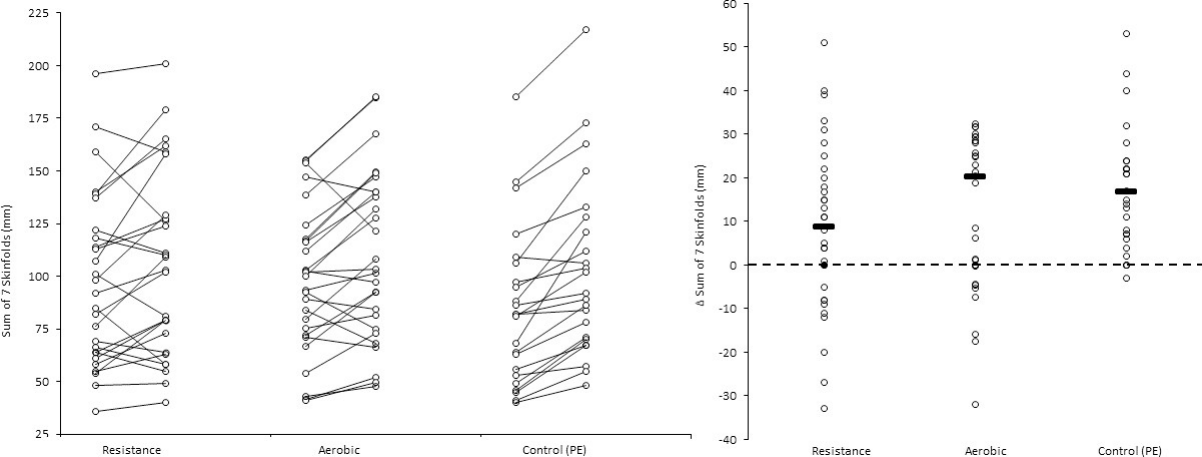
**Left panel: Sum of skinfold difference (mm) pre- and post- intervention for each participant, and group means.** Right panel: Individual participant and mean within-group post-intervention sum of skinfold difference (mm). Pairwise effect sizes for within-group difference: Resistance; *d* = 0.25, aerobic; *d* = 0.40, PE (control); *d =* 0.58.

**Fig 3:**
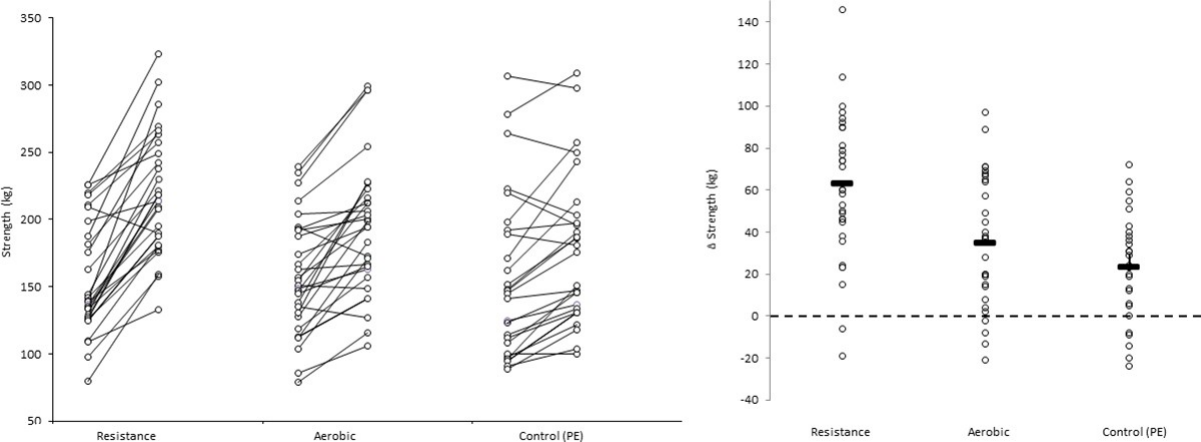
**Left panel: Strength (kg) values pre- and post-intervention for each participant, and group means.** Individual values and mean for within-group difference in strength Right panel:Individual participant and mean within-group post-intervention strength difference (kg). Pairwise effect sizes for within-group difference: Resistance; *d =* 1.2, aerobic; *d* = 0.58, PE (control); *d =* 0.32.

**Fig 4:**
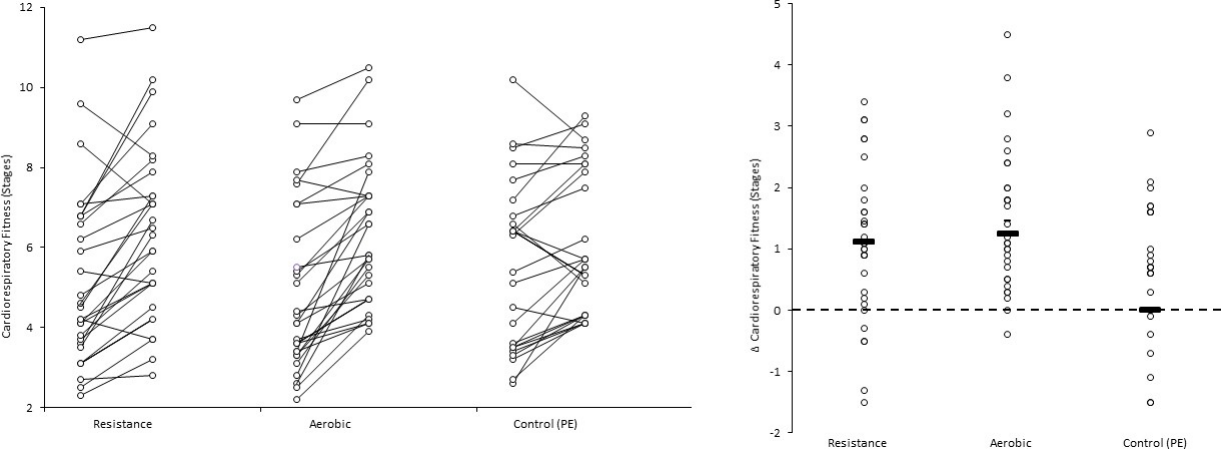
**Left panel: Cardiorespiratory fitness (stages completed on the 20 meter shuttle-run test) pre- and post- intervention for each participant, and group means.** Right panel: Individual participant and mean within-group post-intervention cardiorespiratory fitness difference post- intervention. Pairwise effect sizes for within-group difference: () resistance; *d =* 0.48 aerobic; *d =* 0.58, PE (control); *d =* 0.30.

In addition, for illustrative purposes, we plotted individual and mean pre- and post-test values as Firework Plots (Figs 1–4) and individual and mean difference shown as Dot Plots. The magnitude of within-group differences (pre- to post-test) was expressed as effect size using Cohen’s paired ‘*d*’ where the mean difference is divided by the mean of both standard deviations.

d=m1‐m2/(σ1+σ2/2)

## Results

**Fig 5:**
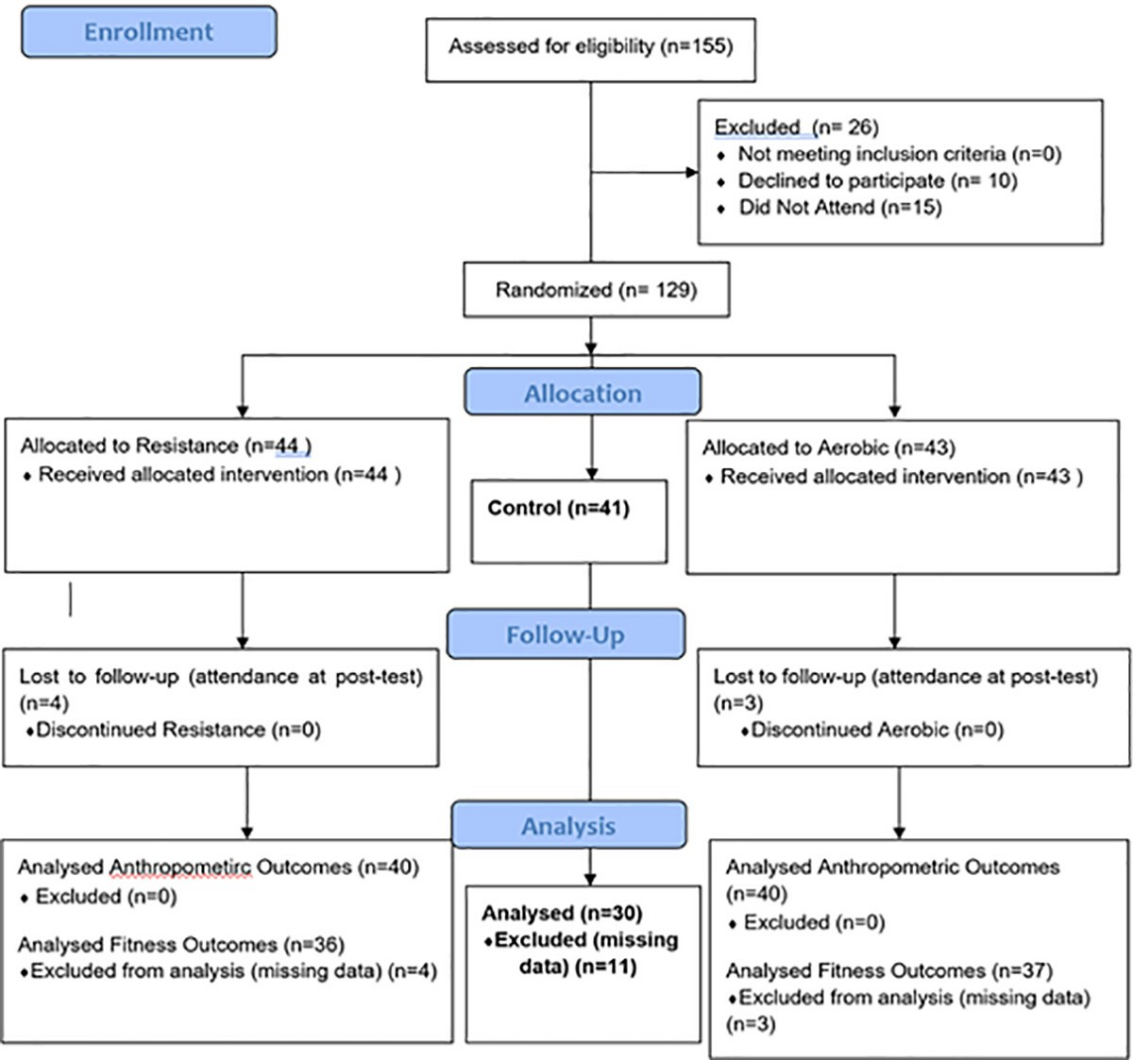
Consort 2010 flow diagram.

Fig 5 shows enrollment, allocation, follow and analysis within the study. Ten of the 120 participants initially randomized were lost to follow-up. Complete anthropometric pre-test and post-test data were available for 110 participants: 40 in the resistance, 40 aerobic and 30 PE (controls). Table 2 shows the anthropometric and fitness characteristics in each group at baseline. Fitness test data were missing for seven participants, 4 resistance and 3 aerobic, with no difference in attendance levels between the two groups. Median attendance was 18 the same for resistance (IQR10-26) and aerobic (IQR 13–27) based on all participants who attended post-test.

**Table 2:** Pre-test characteristics of n = 110 participants with complete data at post-test.

	Resistance (n = 40)	Aerobic (n = 40)	Control (n = 30)
Sex	22 Males, 18 Females	19 Males, 21 Females	15 Males, 15 Females
Age (years)	15.0±0.95	14.8±1.04	14.7±1.09
Body mass (kg)	54.2±9.6	50.7±8.7	50.9±9.2
Stature (cm)	162.1±9.0	160.6±8.1	162.8±8.8
BMI	20.5±3.0	19.9±3.6	19.4±2.6
Body fat (%)	20.1±8.6	19.6±8.9	18.7±7.1
Lean body mass (kg)	42.9±6.8	40.3±4.5	41.2±7.4
SSF (mm)	97.8±47.8	98.5±42.7	96.1±43.8
Strength (kg)	171.2±65.4	145.2±41.7	158.0±62.1
CRF (Stages)	5.6±2.3	5.0±2.0	5.6±2.3

Body fat (%) and lean body mass derived from bioelectrical impedance analysis. SSF: Sum of 7 skinfolds (triceps, biceps, subscapular, suprailiac, abdominal, thigh and calf). Strength: Sum of 6 repetition maximum (kg) in leg-press, bench press and lat pull-down. CRF: Cardiorespiratory fitness expressed as stages (and shuttles) reached on the 20 meter shuttle-run test.

Table 3 shows the results of the ANCOVA comparing adjusted post-test means for each condition and the effect size differences between resistance v aerobic and resistance v control.

**Table 3:** Effectiveness of resistance and aerobic training compared to PE (controls). Estimated marginal means adjusted for baseline values (standard error).

	Condition			Effect Size (95%CI)	
				for difference for mean difference between resistance group.	
	PE (Control)	Aerobic	Resistance	Resistance *vs*. Control	Resistance *vs*. Aerobic
Lean body mass (kg)	42.5 (2.2)	42.6 (2.1)	42.7 (2.0)	*d* = 0.04 *(-0*.*10–0*.*16)*	*d* = 0.03 *(-0*.*12–0*.*08)*
Sum of skinfolds (mm)	122.7 (4.4)	114.4 (3.7)	108.3 (3.6)	*d* = 0.27 *(0*.*09–0*.*36)*	*d* = 0.07 *(-0*.*09–0*.*16)*
Strength (kg)	183.1 (7.9)	194.3 (6.8)	245.3 (6.6)	*d* = 0.66 *(0*.*49–0*.*86)*	*d* = 0.38 *(0*.*28–0*.*67)*
CRF (stages)	6.0 (0.19)	6.7 (0.22)	6.6 (0.2)	*d* = 0.24 *(0*.*10–0*.*36)*	*d* = 0.04 *(-0*.*10–0*.*12)*

Sum of 7 skinfolds: Triceps, biceps, subscapular, suprailiac, abdominal, thigh and calf. Strength: Sum of 6 repetition maximum (kg) in leg-press, bench press and lat pull-down. CRF: Cardiorespiratory fitness expressed as stage (and shuttles) reached on the 20 meter shuttle-run test.

For lean body mass, the main effect for condition was not significant (*F* = 0.44, *P* = 0.89). Differences between resistance and PE (control) and between resistance and aerobic were trivial. For the sum of skinfolds, there was a significant main effect for condition (*F* = 3.43, *P* = 0.04). There was a greater increase in the sum of skinfolds (small magnitude difference) after PE (control) than resistance (*d =* 0.27), while changes in resistance and aerobic did not differ. For strength, there was a significant main effect for condition (*F* = 18.2, *P* < .001). Resistance was more effective (moderate magnitude difference) in increasing strength than PE (control) (*d =* 0.66) and aerobic (*d =* 0.55). For cardiorespiratory fitness, there was a main effect (*F* = 18.2, *P* < .001). Resistance was more effective (small magnitude difference) than PE (control) (*d =* 0.24) in increasing cardiorespiratory fitness, but not more effective than aerobic.

### Within-group changes during intervention

For illustrative purposes, within-group data is shown for each outcome and condition, the left panel of Figs 1–3 shows individual values pre- and post- intervention, the right panel shows individual changes pre v post intervention.

## Discussion

The main aim of the study was to compare the effects replacing 2-hours weekly state school PE class with a twice weekly 16 week after-school supervised resistance training program on health-related fitness and body composition. We also compared resistance training with a simultaneous isotemporal aerobic training program in order to control for the potential effect of supervision and isolate resistance training-specific effects. Participants attended a median of 18 sessions of resistance training (comparable to aerobic attendance) representing only half the number prescribed and equivalent to one session (of < 1 hour) per week. Yet, the benefits on health-related fitness and adiposity of resistance were significantly better than those of a 2 hour standard PE class, and equal or greater than the supervised “control” aerobic training program. Post-intervention analysis adjusting for baseline values showed that relative to PE, resistance training increased strength (*d* = 0.66), improved cardiorespiratory fitness (*d* = 0.24) and attenuated gains in subcutaneous adiposity (*d* = 0.27).

Resistance training led to similar improvement in cardiorespiratory fitness and attenuation of adiposity gains as that observed in the aerobic training group, but to greater strength development. The effects of aerobic training were more specific and did not produce a significant effect on strength. Our results suggest that resistance training could therefore be considered the most effective and time-efficient stimulus for improving three key components of health-related fitness. Strength is lower in both children and adults from low-middle income countries [8, 11] and we previously observed in a similar population of Colombian youth that strength was a better predictor of cardio-metabolic risk profile than cardiorespiratory fitness [3]. Taken together, these suggests that the additional muscular strength gains from resistance training represent a stimulus that should be prioritised in physical activity programs aimed at improving physical health within this population, and potentially other low-middle income countries.

Most PA interventions reported in the literature originate from of high-income countries with a high prevalence of obesity in youth. Commonly the focus of these is the efficacy of aerobic activity to prevent or treat overweight and obesity [15]. Obesity is less prevalent amongst children of low-middle income countries such as Colombia where children from lower-middle social strata remain at risk of marginal nutrition [20]. In the present sample, recruited irrespective of weight status, 89% of participants were either normal weight or underweight with only 2% classified as obese. Paradoxically however, the prevalence of obesity for Colombian adults from this socio-economic stratum is relatively high [20] and higher than in more affluent communities [28], leading to a disproportionate burden of chronic disease [29]. Based on the concept of ‘primordial prevention’ maintaining healthy weight in childhood and adolescence is a key component of obesity prevention [17]. Considering the increase in skinfold thickness of the PE group (Fig 2, Panel B) and reported elsewhere [30], attenuating gains in adiposity appears an appropriate target for an intervention. The reported effect of resistance training on adiposity are somewhat inconsistent [31–33]. A meta-analysis of resistance training interventions observed small but beneficial effects measures of body fat (%) and skinfold thickness in youth [15]. During the present study, BMI changes were trivial with little difference between intervention and control groups, indicating that the measure was not able to detect to exercise induced improvements in body composition identified by skinfolds and bioelectrical impedance analysis.

Despite baseline differences, weight gains followed parallel trajectories in all groups, a pattern also observed for height. The latter is an important finding in the context of the concern amongst some parents that resistance training “may stunt growth” in children, a previously highlighted misconception [34], and a perception that the potentially marginal protein intake of these young people would limit their ability to respond to the resistance program. Instead, participation in resistance training specifically limited gain in adiposity without restraining linear growth or lean body mass gain. Furthermore, while there were trivial post-intervention differences between resistance and PE or aerobic groups in lean body mass, in contrast with the PE and the aerobic group, none of the participants in the resistance training group experienced a decrease in lean body mass (Fig 1, Panel B). This aligns with evidence that resistance training provides a stimulus to improve efficiency of protein absorption and utilisation to conserve muscle and whole-body protein [35]. A 2 kg increase in fat free mass was reported in 15-year-old boys who completed eight weeks of bi-weekly resistance training [31]. In schoolchildren aged 10–14 years, Meinhardt et al. [32] found a small increase in lean body mass of boys after completing 19 weeks resistance training although changes were not significantly different from those of controls. An inadequate intervention period and lower androgen levels in studies which included youth at earlier pubertal stage may explain the lack of consistency of lean body mass / fat free mass gain as an outcome, and overall lack of significant effect in a recent meta-analysis [15].

As might be expected, the largest strength gains were observed in the resistance training group, superior to both PE (*d* = 0.66) and aerobic (*d* = 0.38). Within group increases in strength were similar in the aerobic and PE groups, suggesting that the aerobic training did not accelerate strength beyond growth and maturation related development. Low strength in adolescence is associated with a poorer cardiometabolic profile [2, 3, 36], and is predictive of higher cardiovascular risk factors in young adulthood [4] and a greater risk of all cause and cardiovascular mortality in later life [37]. The positive effects on strength are particularly important in low-middle income countries where muscle strength is reported to be lower [8, 11, 12] and potentially contributes to their populations’ greater susceptibility to chronic disease at a given BMI relative to high-income populations [38]. Based on substantially lower values in Colombian [11, 12] than European youth [13], it also appears that these strength deficits may manifest early on in life. Furthermore, in Colombian youth strength values are lower in lower socioeconomic status when adjusted for differences in body dimensions [12], suggesting that implementing resistance training as a primordial countermeasure in this demographic may have particular value. We previously noted a stronger association between muscle strength and metabolic risk than between aerobic fitness and metabolic risk in a similar population of Colombian schoolchildren [3], whereas in high income countries associations with aerobic fitness are reported to be similar [2] or stronger [39]. Therefore, ethnic/regional differences in the relative influence of muscle strength/resistance exercise on cardiometabolic risk may exist. In addition to associations with cardiometabolic risk profile, improvements in strength may enhance children’s confidence and competence in physical abilities resulting in enhanced physical literacy, self-efficacy and involvement in sports and other physical activity [6, 14]. Indeed, resistance training participation has been shown to lead to increased spontaneous daily physical activity, particularly in the least active children [32].

Aerobic fitness is associated with a more healthful cardiometabolic profile, independently of muscle strength and physical activity [2]. Improved aerobic fitness is considered an important outcome measure for activity interventions [17]. Importantly, while resistance training was more effective than PE (control) in improving cardiorespiratory fitness (*d* = 0.24), its effect on cardiorespiratory fitness was similar to that of the aerobic group (*d* = 0.04). Improvements in aerobic fitness have been reported following resistance training, with a moderate effect size increase in VO_2_ peak (L.min) observed in prepubertal boys who participated in a 12-week resistance training intervention, compared to no change in controls [33].

There was a large number of missed sessions in both intervention groups, but no difference between resistance and aerobic programs. In addition to family and health issues (including an outbreak of Zika) cited by students, parents and school staff as the main reasons for this, the program being provided after-school and off-site may also have created a barrier to attendance. This design was however necessary due to lack of space at the school and the structure of the Colombian state-school day, consisting of two cohorts of students in classes from 6.15 to 12.15 or 12.30 to 6.30, which maximizes the use of the building, but creates a further barrier to in-school programs as neither cohort have a lunch break, just a short recess.

### Limitations and strengths

Poor program adherence is the major limitation of the present study, although there was no evidence of baseline differences and the data appears to be missing at random. It may be however, that those who stayed in the program may have been responders and remained as they were more motivated by strength gains. The missing of sessions means that our analysis, based on intention to treat, underestimates the true impact of a twice weekly resistance (or aerobic) stimulus. While we assumed full attendance of controls in their compulsory curriculum on-site control, we did not record their attendance and therefore cannot verify this. A further limitation of the study was the lack of characterization of effort during the resistance training, which may have allowed more precise quantification of intensity via the use of both repetition maximum and self-perceived effort in testing and prescription [24]. However, given the novelty of the resistance stimulus within this population and the limitations with equipment and time, our aim was to deliver “a general resistance training stimulus” rather than evaluate a specific program. Therefore, these results may not be generalizable across resistance training programs or to youth populations with greater resistance training experience. Resistance training programs are under-investigated generally and more so in the population we studied: low-medium socioeconomic status, low-middle income country youth. As there are ethnic, regional and nutritional influences specific to the present population, these results may not be generalizable to other middle- or low-income countries. There is a need for further research in other low-middle income countries, particularly those identified as having relative strength or muscle mass deficits.

We substituted 1 x 120 min weekly PE class with 2 x 60 min per week resistance training in low-middle socioeconomic status schoolchildren from Colombia, a middle-income country. Resistance training resulted in greater muscular strength and higher cardiorespiratory fitness when compared with PE class. Improvements in cardiorespiratory fitness were similar to that of the twice-weekly aerobic training program while strength gains were superior. Resistance training also attenuated increases in subcutaneous adiposity relative to that observed in PE. Resistance training was effective in improving both aspects of fitness and components of body composition. Combined with epidemiological evidence suggesting that while the present population has both lower cardiorespiratory fitness [40] and strength [11, 12], but have a relatively larger strength deficit than youth in high income countries, a higher priority should be given to reversing the historical under-promotion of resistance training in youth and providing access to resistance training programs to youth in Colombia and other countries with a similar profile. Nonetheless, more research is needed to evaluate means to deliver the resistance training stimulus within the school setting, such as integrating it within PE class, or before/after school. Offsite programs such as the ones evaluated in the current study presented adherence issues, and involved substantial space and equipment requirements, making scalability questionable. There are also two potential concerns around our intervention model in which offsite training replaced curriculum PE. Firstly, it meant that participants who missed the offsite session, missed out completely on structured physical activity during the week. Secondly, while we have shown that participation in a median of 18 resistance training sessions during a school semester were superior to PE in terms of development of cardiometabolic health-related components of fitness and body composition, there is a potential concern that the development of other aspects of physical literacy could be negatively affected if resistance training replaced PE class. It is important to note however that the study was designed to isolate the effect of resistance training, not to promote such a model. Further research should investigate the feasibility of integrating “a dose” of resistance training within PE classes or during another part of the school day within this population, particularly as benefits were observed following the equivalent of a participation in only a single session per week.
